# Protocol for Metagenomic Virus Detection in Clinical Specimens^1^

**DOI:** 10.3201/eid2101.140766

**Published:** 2015-01

**Authors:** Claudia Kohl, Annika Brinkmann, Piotr W. Dabrowski, Aleksandar Radonić, Andreas Nitsche, Andreas Kurth

**Affiliations:** Robert Koch Institute, Berlin, Germany

**Keywords:** viruses, infectious diseases, emerging infectious diseases, virus detection, metagenomics, next-generation sequencing, NGS, metagenome, zoonoses, random PCR, Sendai virus, vaccinia virus, influenza virus, reovirus

## Abstract

Sixty percent of emerging viruses have a zoonotic origin, making transmission from animals a major threat to public health. Prompt identification and analysis of these pathogens are indispensable to taking action toward prevention and protection of the affected population. We quantifiably compared classical and modern approaches of virus purification and enrichment in theory and experiments. Eventually, we established an unbiased protocol for detection of known and novel emerging viruses from organ tissues (tissue-based universal virus detection for viral metagenomics [TUViD-VM]). The final TUViD-VM protocol was extensively validated by using real-time PCR and next-generation sequencing. We could increase the amount of detectable virus nucleic acids and improved the detection of viruses <75,000-fold compared with other tested approaches. This TUViD-VM protocol can be used in metagenomic and virome studies to increase the likelihood of detecting viruses from any biological source.

## Abstract 2

This protocol can rapidly and reliably detect viruses during disease outbreaks and for detection studies.

Viruses responsible for disease outbreaks in humans naturally emerge either from the human population or as zoonoses by transmission from animal hosts (*1*). Viruses can also emerge unnaturally, either directly (e.g., bioterrorist attacks) or accidentally (e.g., laboratory infections). Despite these possibilities of virus emergence, 60% of emerging viruses have a zoonotic origin, thus highlighting transmission from animals to humans as a major threat to public health (*2*). Whenever viruses emerge, prompt identification of the agent and implementation of control measures to contain the outbreak are required.

Currently, various next-generation sequencing (NGS) approaches provide solutions for detection of purified and concentrated viruses (i.e., from cell culture). However, for clinical specimens, such as blood, other fluids, or infected organ tissues, successful detection of viruses is less likely because virus-to-host genome ratios are insufficient (*3*–*6*). Use of tissues from persons with suspected infections for virus detection enables elucidation of infection directly at the site of viral replication. Although detecting viruses directly from infected organ tissue provides obvious and valuable advantages, reliable purification of viruses directly from tissues still remains a challenge.

In this study, we quantifiably and extensively compared classical and modern experimental approaches for virus purification and enrichment to finalize a protocol for unbiased detection of emerging viruses directly from organ tissues (tissue-based unbiased virus detection for viral metagenomics [TUViD-VM]) for an increased signal-to-noise ratio (ratio of virus genome to host genome) in virus detection. Use of this approach will reduce the amount of host nucleic acids required and save money and time in preparation of samples for NGS and the subsequent bioinformatic analysis.

## Materials and Methods

**Figure 1:**
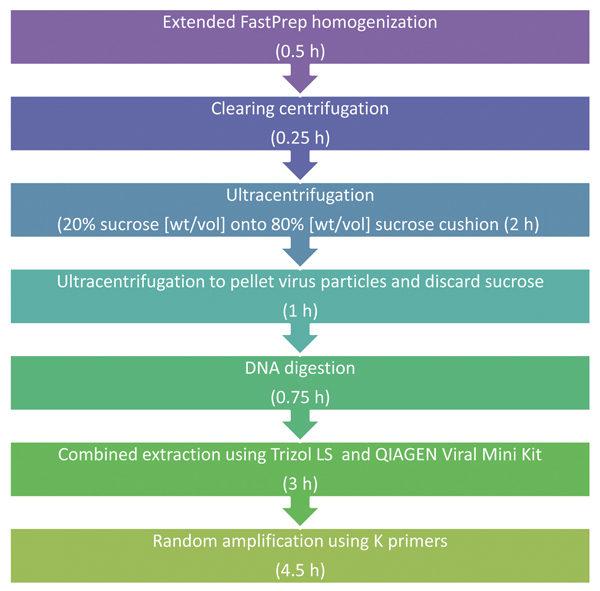
Schematic description of tissue-based universal virus detection for viral metagenomics protocol. Estimated durations of each step are shown in parentheses. The protocol takes 12 h to complete.

We first describe how the protocol was developed and evaluated, We then describe the final virus purification and enrichment TUViD-VM protocol for metagenomic deep sequencing for nucleic acid from organ tissue (Figure 1).

### Protocol Development

#### Ethics Statement

All procedures regarding the marmoset used in this study were performed in accordance with the European Association of Zoos and Aquaria Husbandry Guidelines for Callitrichidae, 2nd ed., 2010 (http://www.marmosetcare.com/downloads/EAZA_HusbandryGuidelines.pdf), which promotes the highest possible standard for husbandry of zoo animals. The marmoset was kept in Zoo Heidelberg (Heidelberg, Germany) with other marmosets in a species-appropriate environment enriched with material for occupation and activity and adequate feeding regimens 3 times a day. The marmoset that was euthanized did not have any additional signs of illness or infection. The production of specific pathogen–free eggs (VALO BioMedia GmbH, Osterholz-Scharmbeck, Germany) was performed in accordance with guidelines of the European Pharmacopoeia (EP7.0.5.2.2) and the US Department of Agriculture Veterinary Services (Memorandum 800.65).

All procedures regarding embryonated chicken eggs were based on German Animal Protection Laws. For infection, fertilized chicken eggs at embryonation day 11 were inoculated with virus into the allantois sack or onto the chorioallantoic membrane. Development of embryos was terminated at day 17 of embryonation by cooling the eggs overnight at 4°C. No further specific approval is needed for experiments on embryonated avians before time of hatching. However, additional approval from the internal ethics advisory board of the Robert Koch Institute was obtained and is available on request.

#### Study Design

To compare classical and modern experimental approaches of virus purification and enrichment, we designed a tissue model for internal organs of chicken, each infected with 1 of 4 viruses (poxvirus [vaccinia virus], reovirus[orthoreovirus], orthomyxovirus [influenza virus], and paramyxovirus [Sendai virus]) at low concentrations (Table 1; Table 2; Technical Appendix). Viruses were chosen on the basis of their role in emerging zoonotic diseases and their morphologic and molecular heterogeneity to obtain results for a broad range of viruses (Table 3).

**Table 1:** Comparison of methods used to develop a protocol for metagenomic virus detection in infectious disease settings*

Purpose, method and supplier	Score†
Virus release/ homogenization	
Ultrasonic (Sonopuls; Bandelin Electronic, Berlin, Germany)	+2
Dounce homogenizer (Kleinfeld Labortechnik, Gehrden, Germany)	+1
Qiashredder (QIAGEN, Hilden, Germany)	0
Trypsin (Life Technologies, Darmstadt, Germany)	+3
FastPrep Homogenizer (MP Biomedicals, Strasbourg, France) (longer homogenization time)	+4
Enrichment of virus particles	
Filtration 0.2- µm filter (Merck-Millipore, Temecula, CA, USA)	+4
Filtration 0.45-µm filter (Merck-Millipore)	−2
Fractionated filtration	−1
Durapore polyvinylidene fluoride filter tubes (Merck-Millipore)	+2
With or without clearing centrifugation	+3
Taguchi-optimized centrifugation: 20% sucrose cushion overlaying 80% sucrose cushion and second clearing ultracentrifugation	+4
PEG-It virus precipitation (System Biosciences, Mountain View, CA, USA)	+1
InRichment Virus Reagent Kit I (Analytik Jena AC, Jena, Germany)	−1
Digestion/removal of host nucleotides	
Turbo DNA-free (Ambion, Darmstadt, Germany) 30 min at 37°C with centrifugation	+4
RiboMinus Eukaryote Kit (Invitrogen Life Technologies, Grand Island, NY, USA)	+1
Nucleotide extraction	
QIAamp UltraSens Virus Kit (QIAGEN)	+2
PureLink Viral RNA⁄DNA (Invitrogen Life Technologies)	+1
QIAamp MinElute Virus Spin Kit (QIAGEN)	−1
RTP DNA/RNA Virus Mini Kit (Invitek, Berlin, Germany)	−2
RTP DNA/RNA Virus Ultra Sense (Invitek)	0
NucleoSpin RNA II (Macherey Nagel, Dueren, Germany)	0
NucleoSpin DNA (Macherey Nagel)	+2
Phenol chloroform extraction (Carl Roth GmbH, Karlsruhe, Germany)	+3
TRIzol LS reagent (Life Technologies)	+4
Amplification	
N12 random primer	+3
N10 random primer	+2
WTA‡	+3
WGA	0
K primer‡ (*7*)	+3
3′ locked random primer(*8*)	+1

*WTA, whole transcriptome amplification; WGA, whole genome amplification. †For every relative quantification result that increased the ratio between host and virus nucleic acids, 1 point was assigned (maximum +4 points if the method led to a better detectability for all 4 viruses). For every decrease, 1 point was subtracted (minimum −4 points). ‡WTA and K primer showed similar results. However, when we considered the lower costs and ease of handling of K primers,  we used K primers for this protocol.

**Table 2:** Sample preparation, infection dose, and copy numbers for virus-infected embryonated chickens after 7 d used to develop a protocol for metagenomic virus detection in infectious disease settings*

Virus family	Strain	Infection dose	Virus-positive organ	Virus copy number/100 µL*
*Reovirinae*	T3/Germany/Bat/342/08	10^−3^	Liver	3.47 × 10^3^
*Orthomyxovirinae*	Influenza A/PR/8/1932	10^−6^	Lung	4.94 × 10^4^
		10^−7^	Lung	8.32 × 10^2^
		10^−5^	Liver	8.74 × 10^4^
		10^−4^	Liver	1.66 × 10^2^
*Poxvirinae*	Vaccinia virus	10^−2^	Kidney	1.83 × 10^8^
		10^−2^	Kidney	2.37 × 10^8^
		10^−2^	Kidney	2.38 × 10^8^
		10^−2^	Intestine	2.23 × 10^8^
		10^−1^	Liver	1.92 × 10^8^
		10^−2^	Liver	9.00 × 10^7^
		10^−2^	Kidney	2.35 × 10^8^
*Paramyxovirinae*	Sendai virus	10^−2^	Liver	2.13 × 10^3^
		10^−2^	Liver	2.14 × 10^5^
		10^−3^	Liver	1.86 × 10^5^

*Homogenized tissue with an average volume of 8 mm^3^/ mL.

**Table 3:** Properties of 4 viruses used to develop a protocol for metagenomic virus detection in infectious disease settings*

Property	*Reovirinae*, reovirus	*Orthomyxovirinae*, influenza virus A	*Poxvirinae*, vaccinia virus	*Paramyxovirinae*, Sendai virus
Size, nm, shape	75–85, icosahedral	80–120, spherical, pleomorphic	270 × 350, brick-shaped complex	150–350, spherical, pleomorphic
Buoyant density, g/mL	1.36	1.2	1.23–1.27	1.31
Size genome, kbp	≈23.5	≈13.5	186–192	≈15.5
RNA/DNA	dsRNA	(–) ssRNA	dsDNA	(–) ssRNA
Genome organization	Linear, 10 segments	Linear, 8 segments	Linear, continuous	Linear, continuous
Envelope	No	Yes	Yes	Yes
Replication	Cytoplasm	Nucleus	Cytoplasm	Cytoplasm
Virion assembly	Cytoplasmic inclusion bodies (viral factories)	Cytoplasm	Cytoplasmic factory areas	Cytoplasm
Release	After virus-induced cell death	Budding from cell membrane	Exocytosis, cell lysis	Budding from cell membrane
Sensitivity	Unknown	Cesium chloride, heat, formaldehyde, SDS, ultraviolet light, oxidation compound	Unknown	Cesium chloride, heat, formaldehyde, SDS, oxidation compound

*Virus data were obtained from King et al. (*9*) and Tidona and Darai (*10*). –, negative. SDS, sodium dodecyl sulfate.

#### Model Tissue and Protocol Development

To establish a model tissue, we inoculated specific pathogen–free embryonated chicken eggs with 1 of the prechosen viruses at different concentrations. A detailed description of egg infection and preparation of the model tissue is shown in the Technical Appendix. Reovirus (T3/Bat/Germany/342/08) (*11*) was chosen to represent a nonenveloped virus, orthomyxovirus (influenza A PR/8/1934) and paramyxovirus (Sendai virus) were chosen to represent enveloped viruses with an RNA genome, and poxvirus (vaccinia virus) was chosen to represent an enveloped virus with a DNA genome (Table 3). Viruses in this study were selected to optimize detection of viral zoonotic emerging diseases and possible virus bioterrorism agents.

**Figure 2:**
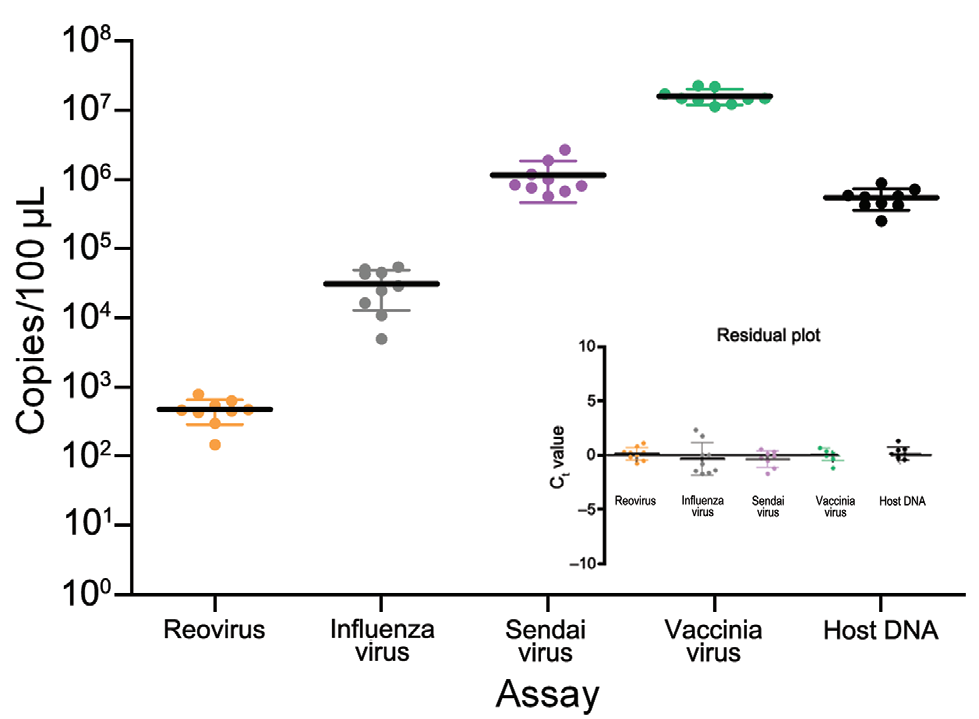
Validation of test aliquots of infected mode used for development of tissue-based universal virus detection for viral metagenomics protocol. Every ninth aliquot was extracted, and viral copy numbers were determined by using a quantitative PCR. Standard deviations (error bars), medians (solid horizontal lines), and residual plots indicate homogeneity and mixture of test specimens. Ct, cycle threshold.

To validate the model tissue homogeneity, we selected every ninth sample for simultaneous RNA/DNA extraction and determined copy numbers for all 4 viruses and the *galTBP* gene (Figure 2). Samples showed an even Gaussian distribution of virus nucleic acids per aliquot and were considered suitable for subsequent experiments.

To establish a protocol for the purification and detection of unknown viruses from animal tissue, we tested different purification techniques and their combinations, including mechanical, enzymatic, and molecular biological methods; the main aim was to eliminate as much host DNA/RNA and maintain as much virus RNA/DNA as possible to optimize random PCR amplification of unknown viruses. The novel established protocol was tested to detect any virus from lung tissue derived from a New World monkey (marmoset), which had to be euthanized because of the unknown disease-causing agent.

We compared different techniques of virus purification, enrichment, and amplification (detailed description of methods compared is shown in the Technical Appendix). In addition, complex purification techniques (digestion and ultracentrifugation) were compared by conducting experiments that had specific control factors (e.g., ultracentrifugation with different concentrations of sucrose, time and speed) (*12*). Organization of combinations of different control factors and their variable factors (e.g., concentration levels, duration or speed in orthogonal assays) enables conducting a minimal number of experiments. On the basis of results of all purification techniques, we developed a combined protocol to provide the maximized yield of virus RNA/DNA after purification.

#### Validation and Analysis of Methods Compared

All compared methods were analyzed simultaneously. Because evaluation of sample quality was ongoing, to exclude any extraction bias, an additional unprocessed control aliquot was extracted and measured with every batch. All results of 1 extraction were rigorously compared with a related control aliquot to normalize any variations caused by extraction, cDNA, and quantitative PCR (qPCR) performance.

Every result was evaluated for increasing the signal-to-noise ratio of virus to host-genome (this ratio is indicated by ). Given that ΔΔx =  Δ measured – Δ control, we assume that the ratio change between virus nucleic acids and host genome is given by ΔΔC_t_  = Δ purified – Δ unprocessed, where C_t_ is the cycle threshold. To visualize relative quantification (RQ), RQ (2 – ΔΔC_t_) was plotted against the respective methods. The RQ value indicates the x-fold change compared with that of the control aliquot (e.g., RQ value of 10 means a 10-fold higher Δbetween virus and host genomes compared with the control aliquot) (*13*). Per definition of the RQ method, the area of significance lays outside RQ values of 0.5 and 2 if the samples show an even Gaussian distribution. Thus, results <0.5 and >2 were considered significant.

An additional scoring system was used to evaluate different methods. For every RQ result that increased the ratio between host and virus nucleic acids, we gave 1 point (maximum +4 points if the method led to better detectability for all 4 viruses), and for every decrease, we subtracted 1 point (minimum is subsequently −4 points). Methods with the highest scores were chosen for establishment of a combined protocol that included purification of unknown viruses from any tissue source (Table 1).

### Final TUViD-VM Protocol for the Enrichment and Purification of Viruses from Organ Tissue

#### Tissue Homogenate

For homogenization, a small cube of tissue (0.5–1 cm^3^) was placed in an autoclaved screw-cap tube (Sarstedt, Hildesheim, Germany) containing 1 mL of phosphate-buffered saline (PBS) buffer and 20–30 sterile ceramic beads. Tissue was disrupted by shaking 4 times at maximum speed at intervals of 15 s by using the FastPrep-24 Instrument (MP Biomedicals, Strasbourg, France). The duration of this procedure was ≈0.5 h.

#### Clearing Centrifugation

A total of 200 μL of homogenate was placed in a 1.5-mL tube and vortexed vigorously. The homogenate was centrifuged for 5 min at 2,000 rpm in a bench top centrifuge (Eppendorf, Hamburg, Germany). The supernatant (≈170 μL) was transferred into a clean tube, and the pellet was discarded. The duration of this procedure was ≈0.25 h.

#### Ultracentrifugation for Virus Particle Separation

A total of 250 μL of 80% (wt/vol) sucrose solution was pipetted into a 2-3/8″ PA ultracentrifuge tube (Beckman Coulter, Krefeld, Germany) and gently overlayed with ≈3 mL of 20% (wt/vol) sucrose solution. The visibility of the phase interface between the 80% and 20% sucrose solutions was checked. The sucrose solution was gently overlayed with cleared tissue supernatant, and PBS was then added to the tubes. The tubes were centrifuged in an SW60 rotor (Beckman Coulter) at 30,000 rpm for 2 h at 4°C. The duration of this procedure was ≈2 h.

#### Ultracentrifugation to Pellet Virus Particles

The layer on the interface between the 20% and 80% sucrose solutions was collected and transferred into a 3-1/2″ tube (suitable for Beckmann SW32Ti rotors; Beckman Coulter). The collected layer was resuspended in ≈40 mL of PBS and mixed gently by pipetting up and down. The suspension was centrifuged for 1 h at 20,000 rpm and 4°C. The supernatant was then discarded. The duration of this procedure was ≈1 h. As an alternative method, virus particles can be precipitated overnight by using Peg-It (System Biosciences, Mountain View, CA, USA).

#### DNA Digestion

The pellet was resuspended in 245 μL of 1× digestion buffer (Turbo DNA Free Kit; Ambion, Darmstadt, Germany). A total of Add 5 μL of Turbo DNase (Turbo DNA Free Kit: Ambion) was added and incubated for 30 min at 37°C. The suspension was transferred to a 1.5-mL reaction tube. A total of 10 μL of stop reagent (Turbo DNA Free Kit; Ambion) wad added, incubated at room temperature for 1 min, and centrifuged at 2,000 rpm for 3 min. The supernatant was transferred to another tube, and pellet was discarded. The duration of this procedure was ≈0.75 h.

#### Combined TRIzol LS Extraction

A total of 750 μL of TRIzol LS (Invitrogen Life Technologies, Grand Island, NY, USA) was added to ≈250 μL of supernatant from previous procedures and homogenized by pipetting up and down 10 times. The mixture was incubated for 5 min at room temperature and centrifuged at 12,000 rpm for 10 min. The supernatant was transferred to precentrifuged phase-lock gel tube (5-Prime, Hilden, Germany). A total of 200 μL of chloroform–isoamyl alcohol was added and mixed by inverting the tube vigorously. The tube was incubated for 15 min at room temperature and centrifuged at 12,000 rpm for 15 min.

Approximately 280 μL of supernatant from the phase-lock gel tube was transferred to another tube containing 1,120 μL of AVL lysis buffer without carrier RNA (Viral RNA Mini Kit; QIAGEN, Hilden, Germany). A total of 700 μL of absolute ethanol was added and mixed by pulse vortexing. The solution was transferred in 600-μL portions to a QIAamp Mini Column, QIAGEN), centrifuged 8,000 rpm for 1 min, and the filtrate was discard. The column was placed in a new collection tube, loaded again, and centrifuged until the lysate was added to the column. A total of 500 μL of 70% (wt/vol) ethanol was added and the column was centrifuged at 8,000 rpm for 3 min.

A mixture of 10 μL of DNase and 70 µL of RDD buffer (RNase-Free DNase Set; QIAGEN) was added to the column and incubated for 15 min at room temperature, as described by the manufacturer. The column was washed with 500 μL of AW1 buffer, centrifuged at 8,000 rpm for 1 min, and the filtrate was discarded. The column was placed in a new tube, 500 μL of AW2 buffer was added, the tube was centrifuged at maximum speed for 3 min, and the filtrate was discarded. The column was then placed in a new tube, and the tube was centrifuged at maximum speed for 1 min to dry the column. A total of 30 μL of elution buffer was added to the column, incubated for 5 min at room temperature, and the column was centrifuged in a new 1.5-mL tube. A total of 30 μL of elution buffer was added to the column, incubated for 5 min at room temperature, and centrifuged in the same tube. RNA (≈60 μL) was chilled on ice. The duration of this procedure was ≈3 h.

#### Random Amplification

Single-stranded cDNA was produced by using the Reverse Transcription Reagent Kit (Applied Biosystems, Foster City, CA, USA) and adapted for a 50-μL reaction containing 30 μL of RNA, 2 μL (40 μmol/L) of K8N random primer (*7*), 3.2 μL (25 mmol/L) of dNTPs, 4 μL 10× buffer, 9 μL (50 mmol/L) of MgCl_2_, 0.8 μL of RNase inhibitor, 0.6 μL of reverse transcriptase, and 0.4 μL of water). A total of 2 μL of K8N random primers and 3.2 μL of dNTPs were added to the 30 μL of RNA and heated at 95°C for 5 min before quenching on ice. The remaining contents of the mixture were heated at 42°C for 60 min before the enzyme was inactivated at 95°C for 10 min.

Double-stranded cDNA was produced by mixing 2 μL of K8N random primers, 3 μL of Klenow buffer (New England Biolabs, Ipswich, MA, USA), and 2 μL (2.5 mmol/L) of dNTPs with 19 μL of cDNA. The mixture was heated at 95°C for 2 min and cooled to 4°C. A total of 1.67 μL of Klenow fragment (New England Biolabs) was added and the mixture was at 37°C for 60 min. Double-stranded cDNA was purified by using the MSB Spin PCRapace Purification Kit (Invitek, Berlin, Germany) and an elution volume of 30 μL. Random amplification was performed by using the procedures reported by Stang and Korn (*7*). Successful random amplification (a 200–2,000-bp smear) was visualized by agarose gel electrophoresis of 10 μL of PCR product. The duration of this procedure was ≈4.5 h. Sequence information can be obtained by either cloning into sequencing vectors or by NGS.

### NGS

RNA samples were fragmented by using the NEBNext Magnesium RNA Fragmentation Module (New England Biolabs). RNA was purified by using RNeasy MinElute (QIAGEN). For cDNA synthesis, Superscript II and Murine RNAse inhibitor (New England Biolabs) were used. Second-strand synthesis was performed by using the NEBNext mRNA Second Strand Synthesis Module (New England Biolabs) and purified by using the MinElute PCR Purification Kit (QIAGEN).

Double-stranded cDNA, DNA, and random PCR products were quantified by using the Qubit HS dsDNA Kit (Invitrogen Life Technologies). Sequencing libraries were established by Ion Xpress Plus Fragment Library Kit (without chemical fragmentation) with indices (Ion Xpress Barcode Adapters 1–16 Kit). The sequencing library was then amplified by using an emulsion-based clonal amplification PCR in the Ion OneTouch 200 Template v2 DL Kit and enriched by using an Ion OneTouch Enrichment System. Sequencing was performed on an IonTorrent PGM in the Ion PGM Sequencing 300 Kit with the Ion 318 Chip Kit (Invitrogen Life Technologies).

### NGS Data Analysis

Programs used for sequence analysis were Geneious Pro R6 (Biomatters, Auckland, New Zealand) and Bowtie2align (*14*). The percentage of bases (Q>20) was ≈80% before length filtering (100–1,000 nt) was applied to remove shorter reads. No additional quality trimming was applied because the quality average was sufficient for our approach. Remaining reads were mapped to the whole reference genomes (or all segments of reference genome) by using Bowtie2align for paramyxovirus (Sendai virus strain Tianjin; GenBank accession no. EF679198), reovirus (T3/Bat/Germany/342/08, 10 segments; JQ412755-JQ412764), orthomyxovirus (influenza H1N1 strain A/Puerto Rico 8-SV14/1934, 8 segments; CY040170-CY040177), and poxvirus (vaccinia virus strain WR, no. AY243312). Coverage of genomes was calculated in weighted average for segmented genomes.

## Results

### Development of Protocol

**Figure 3:**
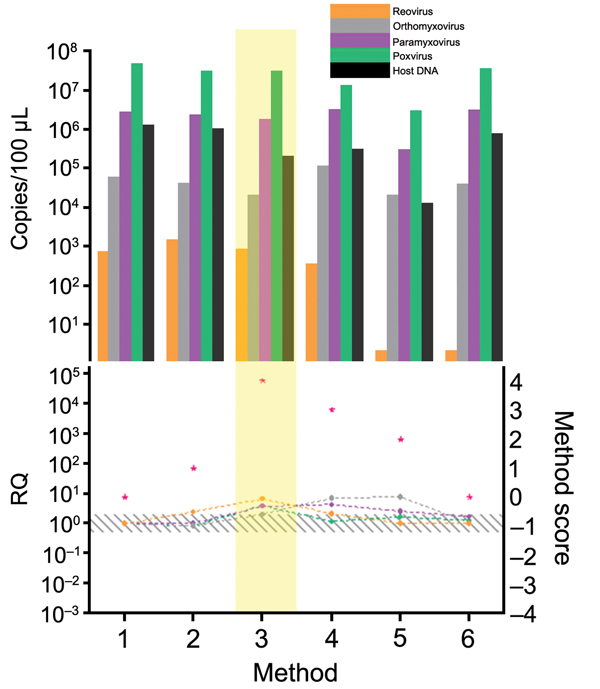
Comparison of tissue homogenization methods used for development of tissue-based universal virus detection for viral metagenomics protocol. Copy numbers were measured by quantitative PCR in duplicate. RQ, relative quantification: RQ (2 – ΔΔCt); (ΔΔCt = Δ purified – Δ unprocessed). Lower panel left y-axis indicates signal-to-noise ratio (RQ) for all viruses tested. The method with the highest score was used to establish the protocol and is shaded in yellow. Red stars indicate highest scores. Diagonally striped area indicates not significant. Ct, cycle threshold. Numbers along baseline indicate method used. 1, control; 2, Dounce homogenizer; 3, extended homogenization; 4, trypsin; 5, ultrasound; 6. QIAshredder (QIAGEN, Hilden, Germany).

**Figure 4:**
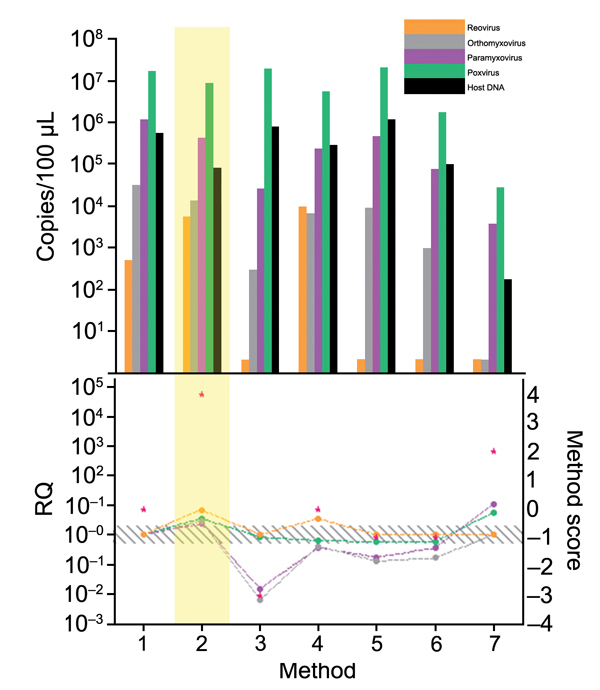
Comparison of filtration methods used for development of tissue-based universal virus detection for viral metagenomics protocol. Copy numbers were measured by quantitative PCR in duplicate. RQ, relative quantification: RQ (2 – ΔΔCt); (ΔΔCt = Δ purified – Δ unprocessed). Lower panel left y-axis indicates signal-to-noise ratio (RQ) for all viruses tested. The method with the highest score was used to establish the protocol and is shaded in yellow. Red stars indicate highest scores. Diagonally striped area indicates not significant. Ct, cycle threshold. Numbers along baseline indicate method used. 1, control; 2, 0.22-μm filter; 3, 0.45-μm filter; 4, filter extraction 1; 5, filter extraction 2; 6, fractionated filtration; 7, filter tubes.

**Figure 5:**
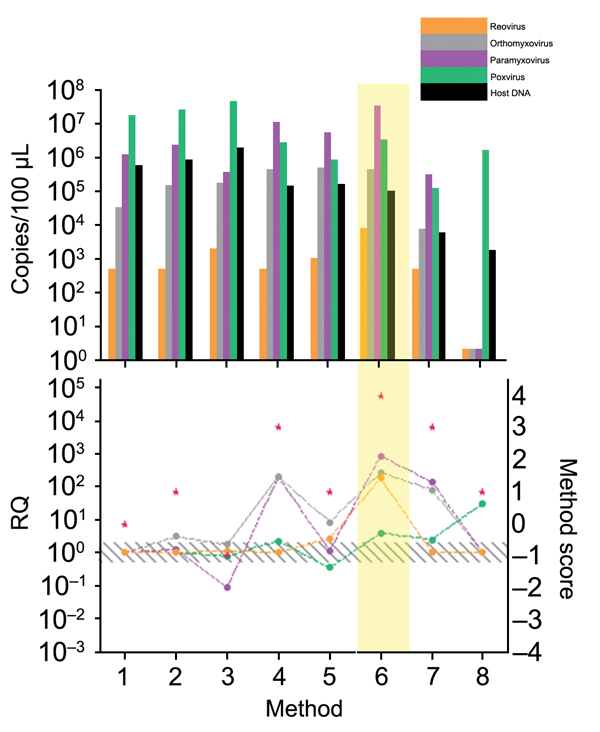
Comparison of enrichment methods used for development of tissue-based universal virus detection for viral metagenomics protocol. Copy numbers were measured by quantitative PCR in duplicate. RQ, relative quantification: RQ (2 – ΔΔCt); (ΔΔCt = Δ purified – Δ unprocessed). Lower panel left y-axis indicates signal-to-noise ratio (RQ) for all viruses tested. The method with the highest score was used to establish the protocol and is shaded in yellow. Red stars indicate highest scores. Diagonally striped area indicates not significant. Ct, cycle threshold. Numbers along baseline indicate method used. 1, control; 2, PEG-It (System Biosciences, Mountain View, CA, USA); 3, InRichment Virus Reagent Kit (Analytik Jena AC, Jena, Germany); 4, clearing centrifugation; 5, clearing centrifugation at 25,000 rpm for 2 h; 6, second clearing centrifugation after 20% sucrose centrifugation; 7, tissue enrichment; 8, Ribominus Eukaryote Kit (Life Technologies, Grand Island, NY, USA).

**Figure 6:**
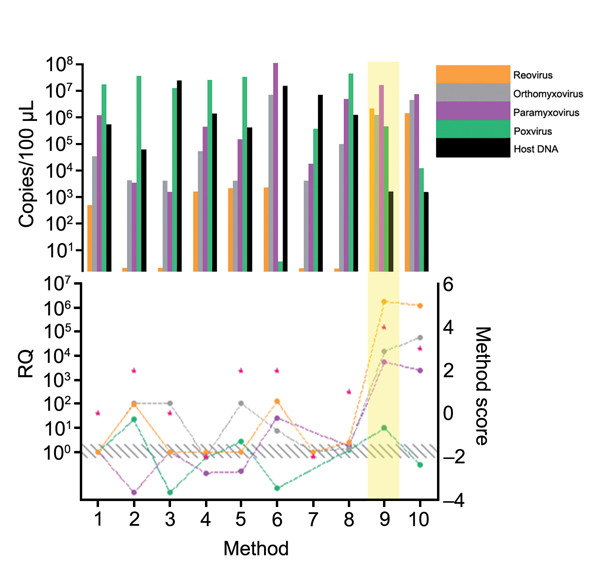
Comparison of extraction methods used for development of tissue-based universal virus detection for viral metagenomics protocol. Copy numbers were measured by quantitative PCR in duplicate. RQ, relative quantification: RQ (2 – ΔΔCt); (ΔΔCt = Δ purified – Δ unprocessed). Lower panel left y-axis indicates signal-to-noise ratio (RQ) for all viruses tested. The method with the highest score was used to establish the protocol and is shaded in yellow. Red stars indicate highest scores. Diagonally striped area indicates not significant. Ct, cycle threshold. Numbers along baseline indicate method used. 1, Nucleospin RNA II (Macherey Nagel, Dueren, Germany); 2, Nucleospin DNA (Macherey Nagel); 3, RTP DNA/RNA Virus Ultra Sense (Invitek, Berlin Germany); 4, RTP DNA/RNA Virus Mini Kit (Invitek); 5, QIAmp UltraSens Virus Kit (QIAGEN, Hilden, Germany); 6, Viral Mini Kit (QIAGEN); 7, QIAmp MinElute Virus Spin Kit (QIAGEN); 8, PureLink Viral RNA/DNA (Invitrogen Life Technologies, Grand Island, NY, USA); 9, TRIzol LS; 10, phenol chloroform.

**Figure 7:**
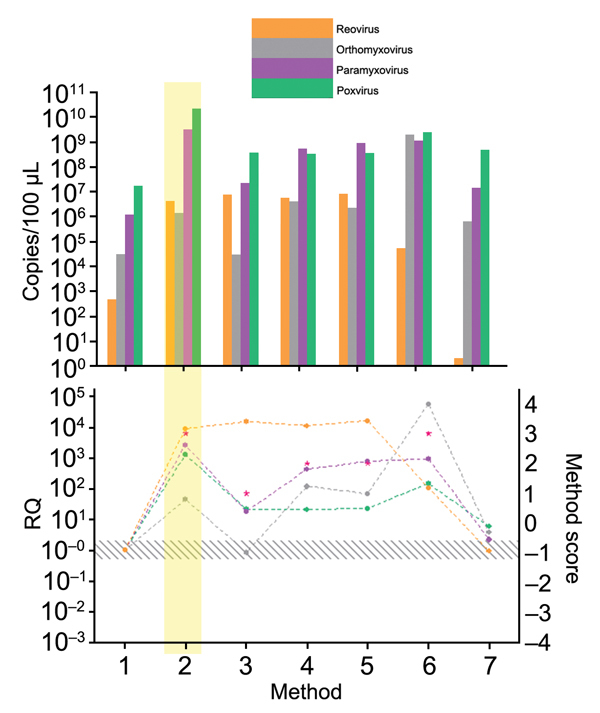
Comparison of primers and random amplification methods used for development of tissue-based universal virus detection for viral metagenomics protocol. Copy numbers were measured by quantitative PCR in duplicate. RQ, relative quantification: RQ (2 – ΔΔCt); (ΔΔCt = Δ purified – Δ unprocessed). Lower panel left y-axis indicates signal-to-noise ratio (RQ) for all viruses tested. The method with the highest score was used to establish the protocol and is shaded in yellow. Red stars indicate highest scores. Diagonally striped area indicates not significant. Ct, cycle threshold. Numbers along baseline indicate method used. 1, control; 2, K primer; 3, 3′ locked primer; 4, N12 primer; 5, N primer; 6, whole transcriptome amplification (QIAGEN, Hilden, Germany); 7, whole genome amplification (QIAGEN).

Every step of the TUViD protocol (homogenization of tissue, filtration, digestion, enrichment, extraction, and random amplification) was compared with alternative approaches. Results are shown in Figures 3,4,5,6,7. Each approach was tested with individual samples, which were measured by using 5 PCRs specific for viruses used and host background in 2 replicates (10 reactions/sample): Results were quantified and evaluated in qPCRs for the 4 viruses and presence of host nucleic acids (Technical Appendix; Table 4; Figures 3,4,5,6,7). A scoring system was developed to assess the optimal combination of all 4 viruses (Table 1; Figures 3,4,5,6,7). A preliminary protocol was further validated and adjusted until no host nucleic acids were detectable by qPCR. This protocol maximized the amount of amplified virus nucleic acids. Subsequently, we established an unbiased protocol for the detection of known and novel viruses in infected organ tissues (TUViD-VM).

**Table 4:** PCRs used to develop a protocol for metagenomic virus detection in infectious disease settings*

Virus or DNA	Characteristic	Primer	Gene, sequence (5′→3′), and reference
Reovirus	PCR target	–	T3/Germany/Bat/342/08 polymerase gene
	Forward primer	BatReoF	CACCATgTCAAgCTgCTCCC
	Reverse primer	BatReoR	ACCgCCATgTATgTCCTCCAg
	Probe	Bat Reo	FAM-CCCAgTCgCggTCATTACCACTCCg-BBQ
	Reference	–	Kohl et al. (*11*)
Influenza virus	PCR target	–	Generic influenza A virus detection
	Forward primer	M+ 25	AgATgAgTCTTCTAACCgAggTCg
	Reverse primer	M-124BB	CCW-gCAAARACATCYTCAAgTYTCTg
	Probe	MGB M+64	FAM-TCAggCCCCCTCAA-MGB
	Reference	–	Schulze et al. (*15*)
Vaccinia virus	PCR target	–	Generic orthopoxvirus detection
	Forward primer	OPV F	TATTACTTCgATTGCTCATCCAgg
	Reverse primer	OPV R	ACTTCTCACAAATggATTTGAAAATC
	Probe	OPV TMGB	FAM-TCCTTTACgTGATAAATCAT-MGB
	Reference	–	Schroeder and Nitsche (*16*)
Sendai virus	PCR target	–	Large polymerase gene
	Forward primer	Sendai F1	gAGCCGCCTggTCTCCTCAgA
	Reverse primer	Sendai R1	AAggCATggCggACAgTggC
	Probe	Sendai MGB	FAM-CCgTAgACAATTACACAAgT-MGB
	Reference	–	In house design
Chicken DNA	PCR target	–	*galTBP* gene NM_205103.1
	Forward primer	galTBP F	gTgTCCACggTgAATCTTgg
	Reverse primer	galTBP R	TgCATTCTAACATACTTCTCTTACCTTg
	Probe	galTBP TM	6-FAM-CgTgCCCGAAATgCTgAATATAATCCCA-BBQ
	Reference	–	Kurth (*17*)
Mammalian DNA	PCR target	–	*c-myc* oncogene HUMMYCF03
	Forward primer	C-myc F	gCCAgAggAggAACgAgCT
	Reverse primer	C-Myc R	gggCCTTTTCATTgTTTTCCA
	Probe	C-Myc TM	Cy5-ATgCCCTgCgTgACCAgATCC-NFQ
	Reference	–	Schroeder and Nitsche (*16*)

*–, not applicable.

### TUViD-VM Validation by NGS

**Figure 8:**
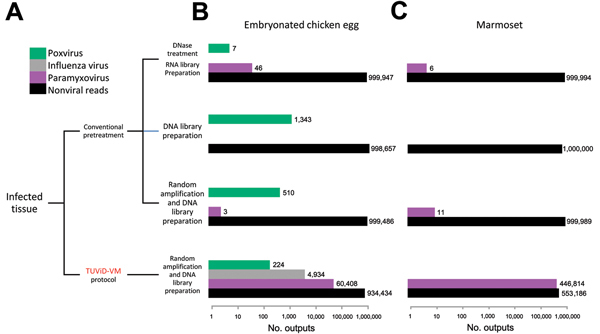
Results of comparative next-generation sequencing used for development of tissue tissue-based universal virus detection for viral metagenomics (TUViD-VM) protocol. A) Sample preparation flowchart to generate 4 next-generation sequencing approaches. B) Results obtained for model tissue (chicken) infected with 4 viruses: vaccinia virus (poxvirus) Sendai virus (paramyxovirus), influenza virus (A/PR8/1934), or reovirus (T3/Bat/G/342/08). The x-axis is log-scaled, and normalized read numbers are indicated. C) Results of marmoset sample proof of principle, Sendai virus–infected lung tissue. The baseline is log-scaled, and normalized read numbers are indicated.

**Figure 9:**
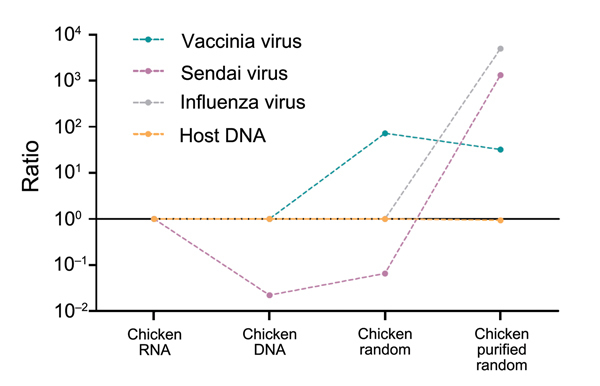
Changes in virus-to-host nucleic acid signal-to-noise ratio during development of tissue-based universal virus detection for viral metagenomics (TUViD-VM) protocol. Next-generation sequencing results for virus-infected chicken tissue comparatively sequenced were obtained by using 4 approaches: standard RNA library preparation (Chicken RNA), standard DNA library preparation (Chicken DNA), DNA library from random-amplified chicken tissue (Chicken random), and DNA library from random-amplified chicken tissue prepared by using TUViD-VM (Chicken purified random). The y-axis is log-scaled.

**Figure 10:**
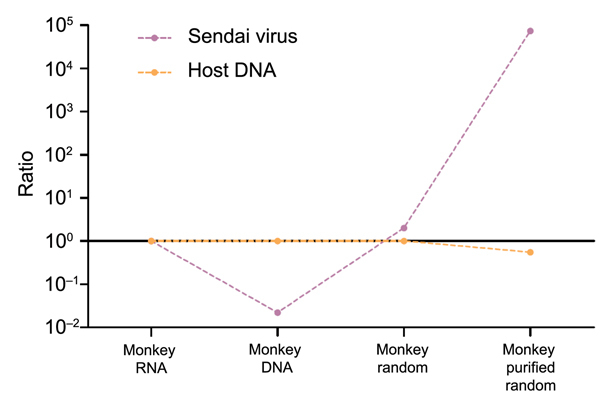
Changes in virus-to-host nucleic acid signal-to-noise ratio during development of tissue-based universal virus detection for viral metagenomics (TUViD-VM) protocol. Next-generation sequencing results for virus-infected marmoset tissue comparatively sequenced were obtained by using 4 approaches: standard RNA library preparation (Monkey RNA), standard DNA library preparation (Monkey DNA), DNA library from random-amplified marmoset tissue (Monkey random), and DNA library from random-amplified marmoset tissue prepared by using TUViD-VM (Monkey purified random). The y-axis is log-scaled.

The TUViD-VM protocol was validated by NGS of 4 aliquots of the model tissue. One aliquot was prepared by using the TUViD-VM protocol developed in this study, and 3 aliquots were prepared by using other approaches commonly used for unbiased virus detection (Figure 8; Technical Appendix). We chose the Invitrogen Life Technologies platform because of its rapid run time and read length, which are crucial for diagnostic purposes. All independent runs were normalized to 1,000,000 output reads for reliable comparison (Table 5; Figure 8). NGS results confirmed the substantial increase in virus nucleic acids, as well as the decrease of host nucleic acids achieved by purification with the novel protocol. The amount of detectable virus nucleic acids was increased >1,000-fold compared with other NGS approaches (Figure 8). For example, although the best NGS approach delivered 40 reads for paramyxovirus in infected chicken tissue, the TUViD-VM protocol resulted in >60,000 reads (97.80% coverage of the complete genome) (Figures 8,9,10; Table 5).

**Table 5:** Output of next-generation sequencing for development of a protocol for metagenomic virus detection in infectious disease settings*

Name	No. original reads	No. remaining reads†	Minimum, maximum (mean) read length), nt†	Sendai virus	Vaccinia virus	Influenza virus, A/ PR8/1934	Reovirus, T3/Bat/G/342/08	No. nonviral reads
Chicken RNA library	1,636,344	1,076,582	100, 464 (240)	46	7	0	0	999,947
Chicken DNA library	1,332,908	808,516	100, 463 (248)	0	1,343	0	0	998,657
Chicken random library	1,347,059	576,467	100, 460 (199)	3	510	0	0	999,486
Chicken TUViD-VM protocol	2,021,403	969,236	100, 455 (220)	60,408	224	4,934	0	934,434
Marmoset RNA library	2,859,201	1,555,567	100, 464 (223)	6	NA	NA	NA	999,994
Marmoset DNA library	2,856,326	1,711,121	100, 464 (246)	0	NA	NA	NA	1,000,000
Marmoset random library	598,451	355,443	100, 464 (200)	11	NA	NA	NA	999,989
Marmoset TUViD-VM protocol	1,007,051	640,088	100, 460 (223)	446,813	NA	NA	NA	553,186

*Standardization value for all procedures was 1,000,000. TUViD-VM, tissue-based universal virus detection for viral metagenomics; NA, not applicable. †After length filtering of 100–1,000 nt.

To provide a proof of concept, we prepared lung tissue from the marmoset that was euthanized and had a natural respiratory infection with Sendai virus by using the 4 approaches and sequenced by using the Invitrogen Life Technologies protocol. Using the TUViD-VM protocol, we found that the amount of detectable virus in marmoset tissue increased 75,000-fold compared with that for other NGS approaches (>400,000 Sendai virus reads compared with 6), which represented 99.98% coverage of the Sendai virus genome and ≈50% of the total read output (Figures 8,10; Table 5).

## Discussion

In this study, we successfully established a purification and enrichment protocol, which shows rapid and reliable results, for detection of known and novel viruses in tissues. Likelihood of detection of RNA viruses was increased. In addition, detection of DNA incorporated in virus particles was not affected even though DNA digestion was performed. The cutoff sensitivity was 100–1,000 virus copies/mL of homogenized organ material (e.g., reovirus; Table 5). The cutoff sensitivity of compared approaches was ≥10^6^ virus copies/mL. The TUViD-VM protocol (from solid tissue sampling to nucleic acid preparation for NGS) takes 12 h to complete. If one allows 16 h for NGS, the TUViD-VM protocol provides sequence data output within 28 h.

Current NGS techniques used for metagenomic approaches produce large amounts of sequence data, which might increase the likelihood of detection of diminutive amounts of virus in comparison with the host genome. The only limiting factor seems to be the cost required for processing 1 sample and capacities for computational analysis of results. This in silico analysis should increase the signal-to-noise ratio of relevant sequences by subtracting nonrelevant sequences, such as the host genome. However, genome sequence data for mammals are limited; only 23 sequences (0.4%) for 5,487 species (*18*). Just 3 genome sequences are available for bats, although they are the second most abundant mammalian species (exceeded only by rodents). There are >1,100 species of bats worldwide and they are suspected vectors of pathogenic viruses (e.g., Ebola virus, Nipah virus, Hendra virus, lyssavirus, and severe acute respiratory syndrome coronavirus). Thus, it seems inefficient to invest large amounts of time, money, and effort in obtaining large datasets, only to invest even more resources to categorize them. Furthermore, quantitative comparison of the virus-enrichment strategies described enables evaluation of multiple classical and modern approaches.

The TUViD-VM described protocol increases the signal-to-noise ratio by as much as 75,000-fold than that for compared approaches and can detect virus genomes quickly in infected tissues (Figures 9,10). Although sequencing of nucleic acid from relatively pure sources (e.g., cell culture, allantoic fluids) is well established and results in reasonable output (*11**,**19**,**20*), sequencing of nucleic acid clinical specimens is still challenging. Other studies reported 0.1% to <10% mammalian virus reads from clinical samples, such as tissue, guano, feces, and pharyngeal swab specimens (*3*,*19*,*21**–**24*). A method reported by Daly at al. showed promising results for detection of DNA viruses but lacked similar results for detection of RNA viruses (*25*). In contrast, our protocol resulted in up to 45% mammalian RNA virus reads directly from infected organ tissue (Figure 8).

After its successful and extensive validation, we highly recommend this protocol for investigation of outbreaks with unknown viral etiologic agents in humans and animals. Furthermore, this protocol can be used in metagenomic virome studies and will be beneficial whenever library construction is necessary (i.e., molecular cloning and NGS) to increase detection likelihood for viruses from any biological source. This protocol would be particularly useful for increasing the signal-to-noise ratio in virus analysis of biological samples in which levels of background nucleic acids are high, which result in difficulties in virus detection and identification. Thus, the TUViD-VM protocol described greatly increases the likelihood of detecting viruses during outbreaks of emerging infectious diseases and in metagenomic virus detection studies.

Technical Appendix Protocol for unbiased virus detection and increasing the signal-to-noise ratios for metagenomics.
