# Supplementary material for: Protocol for Metagenomic Virus Detection in Clinical Specimens
Source: Emerg Infect Dis. 2015 Jan;21(1):48–57. doi: 10.3201/eid2101.140766 (PMC4285256; doi:10.3201/eid2101.140766)
Supplement: Technical Appendix — Protocol for unbiased virus detection and increasing the signal-to-noise ratios for metagenomics. [file 14-0766-Techapp-s1.pdf]

Article DOI: http://dx.doi.org/10.3201/eid2101.140766

Protocol for Metagenomic Virus Detection in Clinical Specimens

Technical Appendix

Protocol for Unbiased Virus Detection and Increasing the Signal-to-Noise Ratios for Metagenomics

Ethics Statement

Production of specific pathogen–free eggs (VALO BioMedia GmbH, Osterholz-Scharmbeck, Germany) was performed in accordance with the guidelines of the European Pharmacopoeia (EP7.0.5.2.2.) and the US Department of Agriculture Veterinary Services (Memorandum 800.65). All procedures regarding embryonated chicken eggs were in accordance with the German Animal Protection Law. For infection, fertilized chicken eggs at embryonation day 11 were inoculated with virus into the allantois sack or onto the chorioallantoic membrane. Development of embryos was terminated at the 17th day of embryonation by cooling the eggs overnight at 4°C. No further specific approval is needed for experiments on embryonated birds before the time of hatching.

All procedures regarding the marmoset were performed in accordance with the European Association of Zoos and Aquaria Husbandry Guidelines for Callitrichidae and promoted the highest possible standard for husbandry of zoo animals. The marmoset was kept in Zoo Heidelberg (Heidelberg, Germany) with other marmosets in a species-appropriate environment enriched with material for occupation and activity and adequate feeding regimens 3 times a day. The dead marmoset did not have additional signs of illness or infection.

Infected Tissue Model/Embryonated Chicken

Reovirus, (bat mammalian orthoreovirus 342/08; T3/Bat/G/342/08) (*1*), influenza virus (A/PR8/1934), paramyxovirus (Sendai virus [SeV]), and poxvirus (vaccinia virus) were provided by the Robert Koch Institute (Berlin, Germany). Fertilized chicken eggs of specific pathogen–free flocks were provided by VALO BioMedia GmbH and incubated at 37°C at a relative humidity of 55%–60%. At day 12 of embryonation, 0.1 mL of serial dilutions of virus stocks

(10^{-1}–10^{-9}) plus 1 negative control (phosphate-buffered saline [PBS]) were inoculated into the allantois sac (T3/Bat/Germany/342/08, flu A PR/8/1934, and SeV) or directly onto the chorioallantoic membrane (vaccinia virus) of the embryonated chicken eggs as described (*2*). Eggs were incubated for 7 days after infection, and survival of the embryo was monitored by candeling. Development of the embryos was terminated at day 17 of embryonation by cooling the eggs overnight at 4°C. Organs (liver, lungs, kidney, spleen, intestine, heart, and gut) were extracted, and each organ was washed in PBS before homogenization.

A tissue cube with an average size of 8 mm^2 was homogenized in 1 mL PBS by using a FastPrep Homogenizer (MP Biomedicals, Strasbourg, France). A total of 0.2 mL of each organ was extracted by using a NucleoSpin RNA II Kit (Macherey-Nagel, Dueren, Germany) without DNA digestion before elution in 60 μL RNAse-free water. cDNA synthesis was accomplished by using a TaqMan Reverse Transcription Reagents Kit (Roche Diagnostics, Mannheim, Germany) according to the manufacturer's instructions. Specific quantitative PCRs (Table 4 in main text) were used for detection of all 4 viruses and a host-specific housekeeping gene (*galTBP*) (*2*). PCRs were performed as described (*1*). Extracted organ tissues were pooled according their viral load (Table 2 in main text). The pooled organ mixture was vortexed vigorously and divided into 150 aliquots (200 μL/aliquot). These aliquots were chronologically numbered from 1 to 150 and stored at –80°C until used.

Compared Methods

Homogenization

To test different homogenization strategies, 5 aliquots (200 μL/aliquot) of model tissue were processed and extracted. An additional control aliquot was extracted at the same time. Tissue was homogenized by using a FastPrep Homogenizer (MP Biomedicals) for 60 s (3 cycles for 20 s at full speed and cooled on ice between cycles) and 120 sec (2 cycles). For enzymatic digestion, 1 aliquot was incubated with 150 μL of trypsin/EDTA (GIBCO Life Technologies, Darmstadt Germany) for 15 min at 37°C. Ultrasonic homogenization was performed by using the SONOPLUS Ultrasound Homogenizer (Bandelin Electronic, Berlin, Germany) in 3 intervals of 1 min in a pulse mode of 5 × 10% with 1 impulse of 1 s divided into a 0.5-s pulse and 0.5-s pause. A Dounce Homogenizer (Kleinfeld Labortechnik, Gehrden, Germany) was used as described according to the manufacturer's instructions. A Qiashredder (QIAGEN, Hilden, Germany) was

used according to the manufacturer's protocol and included 1 centrifugation step at 11,000 rpm for 2 min in a bench-top centrifuge. Only extended homogenization with the FastPrep Homogenizer resulted in slightly increased virus detectability. Thus, we used the extended homogenization time in the final protocol. Results of the compared homogenization steps are shown in Figure 3 of main text.

Filtration

A total of 200 µL of homogenized tissue was filtered by using syringe filters (diameters 0.22 µm and 0.45 µm; Millipore, Cork, Ireland). Fractionated filtration was accomplished by using 0.22-µm and subsequently 0.45-µm syringe filters. Subsequently, used filters were incubated with PBS and extracted to identify possible virus residues. Tissue was also filtered by using low-binding 0.22-µm Durapore polyvinylidene fluoride membrane filter tubes (Millipore). Filtration of tissues resulted in a decreased detectability or deterioration of the ratio between virus and host DNA/RNA. Only a 0.22-µm filter or filter centrifugation tube was able to increase that ratio slightly (Figure 4 of main text).

Digestion

DNA and RNA derived from the host can be digested with different enzymes, whereas nucleic acids inside intact virus particles should be protected by the virus capsid. Enzymatic digestion of the samples with the enzymes Benzonase (Promega, Mannheim, Germany) and RNase A (QIAGEN) and the Turbo DNA-Free Kit (Ambion, Darmstadt, Germany) were tested in 9 experiments according to the procedures of Taguchi (*3*) and Cobb and Clarkson (*4*) Control factors were centrifugation before digestion (30,000 rpm in an ultracentrifuge, 1,000 rpm in a bench-top centrifuge, and no centrifugation); combination of enzymes (Benzonase/RNase/DNase, Benzonase/RNase, and only Benzonase); incubation time (30 min, 60 min, 180 min); and inactivation of the enzyme after the digestion (without inactivation, inactivation with special enzyme inhibitors, and centrifugation at 1,500 rpm). Results of quadratic loss functions are available on request. Optimal conditions for digestion of nucleic acids were calculated by using the method of Taguchi (*3*) (30 min at 37°C, DNase only, centrifugation, and inactivation) and subsequent confirmed in another experiment. This digestion method was included into the tissue-based universal virus detection for viral metagenomics (TUViD-VM) protocol.

Enrichment

Ultracentrifugation

Different centrifugation techniques were tested in 9 experiments. Control factors were the concentration of sucrose (0%, 30%, and 50%); centrifugation speed (15,000 rpm, 25,000 rpm, and 32,000 rpm in an ultracentrifuge); and duration of centrifugation (1 h, 2 h, and 3 h). In addition, 1 sample was centrifuged a second time (30,000 rpm for 1 h). All samples, except 1, were centrifuged (1,000 rpm for 10 min) before ultracentrifugation. We tested increasing the delta value between virus and host nucleic acids. The optimal combination was found to be 30% of sucrose, 2 h of centrifugation at 15,000 rpm or 35,000 rpm. Results of quadratic loss functions and signal-to-noise ratios are available on request. The second ultracentrifugation provided useful results. The optimal parameters (2 ultracentrifugations) were supported by a confirmation experiment and were subsequently used as part of the protocol (Figure 5 of main text). Comparative clear centrifugation was used. For paramyxovirus (SeV) and influenza virus (A/PR8/1934), the signal-to-noise ratio was increased.

Kits

Enrichment was performed by using the InRichment Virus Reagent I Kit (Analytic Jena AC, Jena, Germany). In another enrichment approach, viral particles were precipitated by using 5× Peg-it Virus Precipitation (System Biosciences, Mountain View, CA, USA) overnight according to the manufacturer's protocol. Isolation of pure virus RNA and elimination of tissue-related rRNA was performed by using the Ribo Minus Eukaryote Kit (Invitrogen Life Technologies, Grand Island, NY, USA) and the Ribo Minus Concentration Module (Invitrogen Life Technologies) according to the manufacturer's protocol. The InRichment Virus Reagent I Kit resulted in increased detection of all target nucleic acids, but no improvement of signal-to-noise ratio was observed. The Ribo Minus Kit helped reduce the amount of host nucleic acids, but also reduced the amount of virus nucleic acids and was therefore not used.

Extraction of Nucleic Acids

To evaluate different approaches for nucleic acid extraction, we compared the following commercially available kits according to the manufacturer's protocols and methods: NucleoSpin RNA II (Macherey-Nagel) without DNA digestion, NucleoSpin Tissue Kit (Macherey-Nagel), PureLink Viral RNA/DNA Kit (Invitrogen Life Technologies), QIAmp UltraSens Virus Kit

(QIAGEN), QIAmp MinElute Virus Spin Kit (QIAGEN), Viral Mini Kit (QIAGEN), RTP DNA/RNA Virus Ultra-Sense Kit (Invitek, Berlin, Germany), RTP DNA/RNA Virus Mini Kit (Invitek), classical phenol/chloroform extraction (*5*) (Carl Roth GmbH, Karlsruhe, Germany), and TRIzol LS (Invitrogen Life Technologies). Every kit was tested with 1 of the infected tissue model aliquots. Freshly extracted DNA/RNA was eluted in 60 μL of RNAse-free water (QIAGEN).

Results were analyzed regarding their capability to increase the signal-to-noise ratio between virus and host nucleic acids in comparison to our standard kit (NucleoSpin RNA II, without DNA digestion). Whenever a DNA extraction was suggested in any of the kits, this step was excluded to prevent digestion of virus nucleic acids. The QiaAmp Ultra Sense Virus Kit and the QIAGEN Viral Mini Kit showed the best results within the range of extraction kits. However, the best results for our purpose were shown by extraction using the TRIzol LS. Extraction results are shown in Figure 6 of main text. We combined extraction with TRIzol LS and the QIAGEN Viral Mini Kit in the overall purification protocol.

Random Amplification

Different random amplification primer strategies (K primer [*6*], 3′-locked primer [*7*], and conventional random primers N_{10} and N_{12}) were used. These strategies were compared by using 60 μL of RNA/DNA extracted with the NucleoSpin RNA II Kit (Macherey-Nagel).

Random Primer

For random cDNA synthesis, 60 μL of purified RNA/DNA was incubated with 4 μL (10 μmol/L) of K random primers (*6*) and 6.4 μL (25 μmol/L) of dNTPs for 5 min at 65°C. Reverse transcription of the first strand was accomplished by incubating with 5U of reverse transcriptase, 1.6 μL of RNase Inhibitor (Roche Diagnostics), 8 μL of 10× reaction buffer, 18 μL of 25 mmol/L $MgCl_2$, and 1.2 μL of water for 60 min. The reaction was stopped by incubating at 95°C for 10 min. Second-strand synthesis was accomplished by incubation of 20 μL of first-strand product with 2 μL of 50 μmol/L of K-random primers, 3 μL of 10× Klenow buffer (2 min at 95°C) and 1.7 μL of 25 mmol/L Klenow fragment for 60 min at 37°C. Double-stranded cDNA was amplified by using 5 μL of Klenow reaction product, 4 μL of 10 μmol/L of K primer, 9 μL of Ampli Taq Polymerase (5U/μL), 5 μL of 10× Ampli Taq polymerase buffer (Applied

Biosystems, Darmstadt, Germany), 4 μL (2.5 mmol/L) of dNTPs, 10 μL of 50 mmol/L $MgCl_2$, and 13 μL of water. The PCR mixture was incubated according to the protocol of Stang and Korn (*6*).

The 3′-locked, N10, and N12 random PCRs are used with DNA or cDNA. PCRs were performed by using 5 μL of cDNA, 0.5 μL of either 3′-locked-random-primer (40 μmol/L), N_{10} primer (40 μmol/L) or N_{12} primer (40 μmol/L), 2.5 μL of 10× Platinum buffer (Invitrogen Life Technologies), 1.0 μL (2.5 μmol/L) of dNTPs (Invitrogen Life Technologies), 2.0 μL of 50 μmol/L $MgCl_2$ (Invitrogen Life Technologies), 0.2 μL (5U/μL) of Platinum Taq Polymerase (Invitrogen Life Technologies), and 13.8 μL of water. PCR mixtures were incubated according to the protocol of Clem et al. (*7*).

Kits

Two kits were tested. Freshly synthesized RNA, cDNA, or DNA was amplified by using multiple displacement amplification with the REPLI-g UltraFast Mini Whole Genome Amplification Kit (QIAGEN) and the QuantiTect Whole Transcriptome Kit (QIAGEN) according to the manufacturer's protocol.

Random amplification efficiency of the different methods was evaluated regarding the increase of the signal-to-noise ratio between virus and host nucleic acids (Figure 7 of main text). K primer amplification and the QuantiTect Whole Transcriptome Kit showed comparable results. However, the amount of generated virus-specific nucleic acids was slightly higher when K primers were used. Thus, because K primers are comparable inexpensive and easier to control than commercial kits, this amplification method was used in the TUViD-VM-protocol.

Development of Protocol

To establish a full protocol on the basis of individual experiments, we combined the best methods and tested them with model tissue before evaluating the signal-to-noise ratio. From all single method sets, the best methods were selected and progressively used. The TUViD-TM protocol is shown in Figure 1 of main text. Tested methods were homogenization, filtration, methods for the enrichment of viruses, digestion of background nucleic acids, nucleic acid extraction methods, and random amplification strategies. The different methods tested are described in Table 1 of main text.

References

1. Kohl C, Lesnik R, Brinkmann A, Ebinger A, Radonić A, Nitsche A, et al. Isolation and characterization of three mammalian orthoreoviruses from European bats. PLoS ONE. 2012;7:e43106. PubMed http://dx.doi.org/10.1371/journal.pone.0043106

2. Kurth A. Possible biohazard risk from infectious tissue and culture cells preserved with RNAlater. Clin Chem. 2007;53:1389–90. PubMed http://dx.doi.org/10.1373/clinchem.2007.085894

3. Taguchi G. Introduction to quality engineering : designing quality into products and processes. Tokyo: Asian Productivity Organization; 1986.

4. Cobb BD, Clarkson JM. A simple procedure for optimising the polymerase chain reaction (PCR) using modified Taguchi methods. Nucleic Acids Res. 1994;22:3801–5. PubMed http://dx.doi.org/10.1093/nar/22.18.3801

5. Chomczynski P, Sacci N. Single-step method of RNA isolation by acid guanidinium extraction. Anal Biochem. 1987;162:156–9. PubMed

6. Stang A, Korn K. Characterization of virus isolates by particle-associated nucleic acid PCR. J Clin Microbiol. 2005;43:716–20. PubMed http://dx.doi.org/10.1128/JCM.43.2.716-720.2005

7. Clem AL, Sims J, Telang S, Eaton JW, Chesney J. Virus detection and identification using random multiplex (RT)-PCR with 3′-locked random primers. Virol J. 2007;4:65. PubMed http://dx.doi.org/10.1186/1743-422X-4-65
